# A neurophysiological approach to the distinction between motor and cognitive skills: a functional magnetic resonance imaging study

**DOI:** 10.3389/fnins.2023.1178800

**Published:** 2023-05-19

**Authors:** Yunhang Lu, Jingu Kim, Teri Kim

**Affiliations:** ^1^Department of Physical Education, Kyungpook National University, Daegu, Republic of Korea; ^2^Institute of Sports Science, Kyungpook National University, Daegu, Republic of Korea

**Keywords:** cognitive task, motor task, functional MRI, Gomoku, cognitive load

## Abstract

This study investigated the neurophysiological differences underpinning motor and cognitive skills by measuring the brain activity via functional magnetic resonance imaging. Twenty-five healthy adults (11 women, 25.8 ± 3.5 years of age) participated in the study. We developed three types of tasks, namely, simple motor task (SMT), complex motor task (CMT), and cognitive task (CT), using two-dimensional images of Gomoku, a traditional game known as *five in a row*. When shown the stimulus, participants were instructed to identify the best spot to win the game and to perform motor imagery of placing the stone for the SMT and CMT but not for the CT. Accordingly, we found significant activation from the CMT minus SMT contrast in the dorsolateral prefrontal cortex, posterior parietal cortex, precentral gyrus, and superior frontal cortex, which reflected increased visuospatial attention, working memory, and motor planning. From the CT minus SMT contrast, we observed significant activation in the left caudate nucleus, right medial prefrontal cortex, and right primary somatosensory cortex, responsible for visuospatial working memory, error detection, and cognitive imagery, respectively. The present findings indicate that adopting a conventional classification of cognitive and motor tasks focused on the extent of decision making and motor control involved in task performance might not be ideal.

## 1. Introduction

In the motor-learning and performance domain, various forms of motor tasks, such as discrete, serial, continuous, fine, gross, open, and closed motor skills, are used for experimental testing (Ashford et al., 2006; Grissmer et al., 2010; Schmidt et al., 2018). While the classification of discrete (e.g., throwing), continuous (e.g., swimming), and serial (e.g., gymnastics) motor skills is based on the continuity dimension, open (e.g., soccer) and closed (e.g., golf) motor skills are determined by their perceptual attributes and environmental predictability (McMorris, 2014; Schmidt et al., 2018; Gu et al., 2019). In addition, motor skills can be classified into gross and fine according to the size of the muscles involved (Matheis and Estabillo, 2018).

Another classification of motor skills considers the relative importance of the cognitive elements required for skill execution. Although some motor tasks are commonly compared with cognitive tasks, their boundaries cannot be clearly demarcated. For example, throwing a ball appears to be a simple motor task. However, it requires cognitive processing although the degree of cognitive involvement may vary depending on target presence, distance, size, etc. Thus, cognitive elements are present in all motor tasks (Leisman et al., 2016).

The distinction between cognitive and motor skills is a broad concept, which can be defined in several ways. For example, according to Schmidt and Wrisberg (2004), a cognitive task is one in which decision making is maximized; conversely, a motor task is one in which decision making is minimized. Other researchers have defined motor skills as a set of learned movements that together produce the smooth and efficient movements required to master a specific task (Zeng et al., 2017; Papale and Hooks, 2018). Compared with motor skills, cognitive skills require more information processing, advanced cognition, decision making, and attention. One key characteristic of cognitive tasks is that they take longer than motor tasks because they need time for decision making. Kosinski (2008) argued that compared with simple motor tasks, not only reaction times but also response times slow down when performing cognitive tasks. Thus, although previous researchers attempted to distinguish between the two tasks, their hypotheses lacked concrete empirical evidence (Schmidt and Wrisberg, 2004; Stoodley, 2012).

Different tasks recruit different regions of the brain. While both motor and cognitive tasks are controlled and executed primarily by multiple regions, including the frontal lobe and cerebellum, the primary sensorimotor cortex participates in processing complex bimanual motor tasks (Kermadi et al., 2000). The supplementary motor area (SMA) in the frontal lobe is responsible for transforming a motion sequence into a time sequence, bimanual coordination, and motor learning in complex motor tasks (Seitz et al., 1990; Shima and Tanji, 1998; Meyer-Lindenberg et al., 2002). In addition, the inferior frontal gyrus is involved in controlling the internal timing of motor task planning (Rao et al., 1993). In terms of task difficulty, the primary motor cortex (M1) is more activated during complex motor tasks than during easy motor tasks (Buetefisch et al., 2014). Buetefisch et al. (2014), with the use of functional magnetic resonance imaging (fMRI) to measure changes in M1 activation while performing finger motor-control tasks, observed significantly increased M1 activation with increasing task accuracy. In addition, Boecker et al. (2002) noted a positive correlation between the degree of premotor cortex (PMC) activation and the difficulty of finger-tapping tasks. Shibasaki et al. (1993) identified a significant increase in the activation of auxiliary motor areas and the PM in case of multi-finger cooperative taps. A study on eye- and hand-tracking movements revealed that the cerebellum was not active during solitary hand movements or simple-task eye movements but was significantly activated during coordinated eye–hand tracking (Miall et al., 2001). Therefore, the recruitment of the cerebellum is associated with both cognitive and motor tasks; however, the functional subregions of the cerebellar lobes may play varying roles depending on the task (Stoodley, 2012).

These previous neuroimaging studies did not identify distinctive neurophysiological characteristics between motor and cognitive tasks, but their findings suggest that motor and cognitive tasks may involve different patterns of brain activity. Therefore, the present study aimed to investigate neurophysiological differences underpinning motor vs. cognitive skills by measuring the brain activity during the performance of simple motor, complex motor, and cognitive tasks using fMRI.

## 2. Materials and methods

### 2.1. Participants

Thirty healthy collegiate participants were initially recruited *via* an online advertisement posted at the university website, all of whom participated in the study. However, the results of five participants were contaminated; thus, only the data of 25 participants (11 women, 25.8 ± 3.5 years old) were used for final analysis. All participants were right-handed and were novices or had minimal experience in Gomoku. None of them had a history of neurological disease or psychiatric disorders. All participants provided written informed consent. As compensation, each participant received a cash prize of 50 USD upon completion of their participation. This research was approved by the Institutional Review Board of Kyungpook National University (2022–0032) and conducted in accordance with the Declaration of Helsinki and following a confidentiality agreement.

### 2.2. fMRI scanning

Scanning was performed at the Daegu–Gyeongbuk Medical Innovation Foundation Medical Device Development Center. Brain-imaging data were acquired using a 3.0 T magnetic resonance system (MAGNETOM Skyra; Siemens Healthcare, Erlangen, Germany) and a 64-channel head and neck coil. T1 structural data were acquired using a 3D IR-prepared fast spoiled gradient-echo sequence (BRAVO, TR = 8.5, TE = 3.3, TI = 450, flip of view = 220 mm), with an in-plane resolution of 1 mm isotropic. Echo-planar images were obtained using the following parameters: TR = 3,000 ms, TE = 30 ms, view flip = 220 mm, FOV = 230 mm, matrix = 64 × 64, and slice thickness = 4 mm.

### 2.3. Experimental task

For the experiment, stimuli were developed using two-dimensional images of Gomoku, the traditional game of five stones. When presented with the stimulus on their turn (black stone) to place the stone, the participants were instructed to locate the best spot to win the game. The stimuli included three types of tasks depending on the relative cognitive–motor element of the task: (1) simple motor task (SMT), (2) complex motor task (CMT), and (3) cognitive task (CT). A total of 48 task trials (16 trials for each of the three task types) were presented in a randomized order (Supplementary Figure S1). The SMT was designed to have a minimized cognitive element and required the participants to place the Gomoku stone on either side of four consecutive black stones, creating *five in a row*. For the CMT, the participants were presented with an image of the Gomoku board with four black stones already placed in a row among other stones (total number of stones: 25–35) and asked to place a black stone to make *five in a row*. The CMT was designed in such a way that it required greater cognitive effort than the SMT but an equal motor load. The CT necessitated the participants to locate the most favorable spot in the Gomoku stone to create *five in a row*, with 25–35 stones already placed on the board. Without a motor element, the CT was designed to induce maximal cognitive effort by not including four black stones in a row. Owing to limited body movement within the fMRI scanner, the participants were instructed to imagine the actual physical placement of the stone after deciding where to place the stone during the SMT and CMT, whereas the CT only required them to make decisions about where to place the stone. The task was run using E-Prime 2.0 Professional software according to the protocol depicted in Supplementary Figure S1.

### 2.4. Procedure

Upon arrival to the laboratory, the participants received general information on the study, including the experimental paradigm and the potential risk of participation, after which they voluntarily provided written informed consent. Later, the participants practiced playing Gomoku on a computer to familiarize themselves with the game. Before entering the fMRI scanner, the participants removed all metal objects from their bodies and changed into comfortable clothes. The participants lied down on the scanner in a supine position, with their head cushioned to reduce movement and both head and body immobilized within the chamber. The participants were shown experimental stimuli via MRI-compatible goggles (VisuaStim, Resonance Technology, Inc., Northridge, CA, United States) connected to a Hewlett-Packard portable workstation (screen-resolution 800 × 600, refresh rate 60 Hz). The participants performed a single session of 48 trials, including SMT, CMT, and CT tasks randomly presented 16 times each. The E-Prime log-file automatically saved the stimulus presentation order as it was randomized for each participant to later sort out the task types. The fMRI result files were then classified and categorized according to this order for analysis. The fMRI scanning time was 19 min 18 s, and T1 neuroimaging generation took 6 min 2 s. Overall, the experiment required approximately 25 min 20 s per participant.

### 2.5. Data analysis

Image preprocessing and statistical analysis of the fMRI data were performed using the statistical parametric mapping tool box version 12 (SPM8)^1^ implemented in MATLAB R2019a (MathWorks, Inc., Sherborn, MA, United States). Functional images were preprocessed using conventional preprocessing pipelines: slice-timing correction, head motion correction, spatial realignment, coregistration to the 3D anatomical dataset, spatial normalization to the stereotactic Montreal Neurological Institute space, and spatial smoothing using a Gaussian kernel. The full width at half-maximum of the Gaussian kernel was 8 mm. A high-pass filter of 128 s was applied using the voxel-by-voxel method to remove low frequency drifts in the signal. To obtain parameter images of the contrasts of each condition, the first-level individual analysis of the preprocessed fMRI data used a general linear model with a boxcar hemodynamic response function. The estimated motion correction parameters were included as additional covariates. Subsequently, these first-level contrasts were included in the second-level random effect analysis, which involved a one-sample *t*-test. From the perspective of the exploratory and interpretative nature, the SPM{t}s were thresholded at uncorrected *p*<0.005 for multiple comparisons across the whole brain. Finally, the results of the brain activation maps were projected onto T1-weighted anatomical images.

## 3. Results

**Figure 1:**
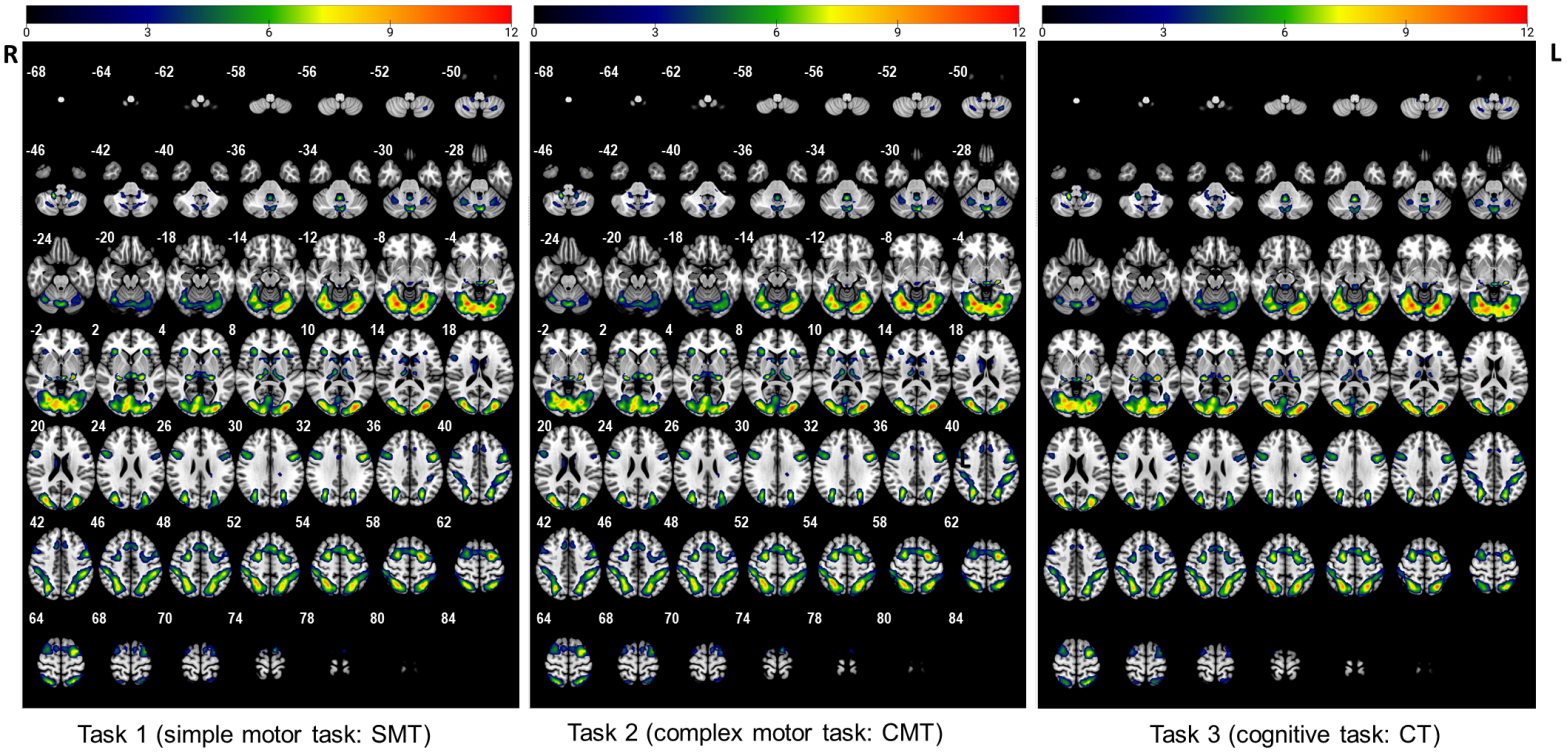
The activated regions during performance of the three tasks (uncorrected: *p* < 0.005, cluster 5).

To determine different neuroanatomical areas activated by the three Gomoku tasks (i.e., SMT, CMT, and CT), we first determined the activated areas for each of the three tasks separately. Activation clusters surviving a voxel-level threshold of *p* < 0.005 are presented in Figure 1 and Supplementary Table S1. Specifically, the SMT engaged significant bilateral activation of the fusiform gyrus (BA19), lingual gyrus (BA18), middle occipital cortex (BA19), superior parietal cortex (BA7), precentral (BA6), and supplementary motor area (BA6). The brain regions activated during the CMT were bilateral fusiform gyrus (BA18), lingual gyrus (BA18), middle occipital cortex (BA18, BA19), superior parietal cortex (BA7), percentral (BA8, BA9), supplementary motor area (BA6), superior frontal cortex (BA6), caudate, thalamus, and insula. The CT was associated with significant activation of the fusiform gyrus (BA18) lingual gyrus (BA 18), middle occipital cortex (BA18, BA19), superior parietal cortex (BA7), precentral (BA8, BA9), supplementary motor area (BA6), superior frontal cortex (BA6), caudate, thalamus, and insula.

### 3.1. CMT minus SMT contrast

**Figure 2:**
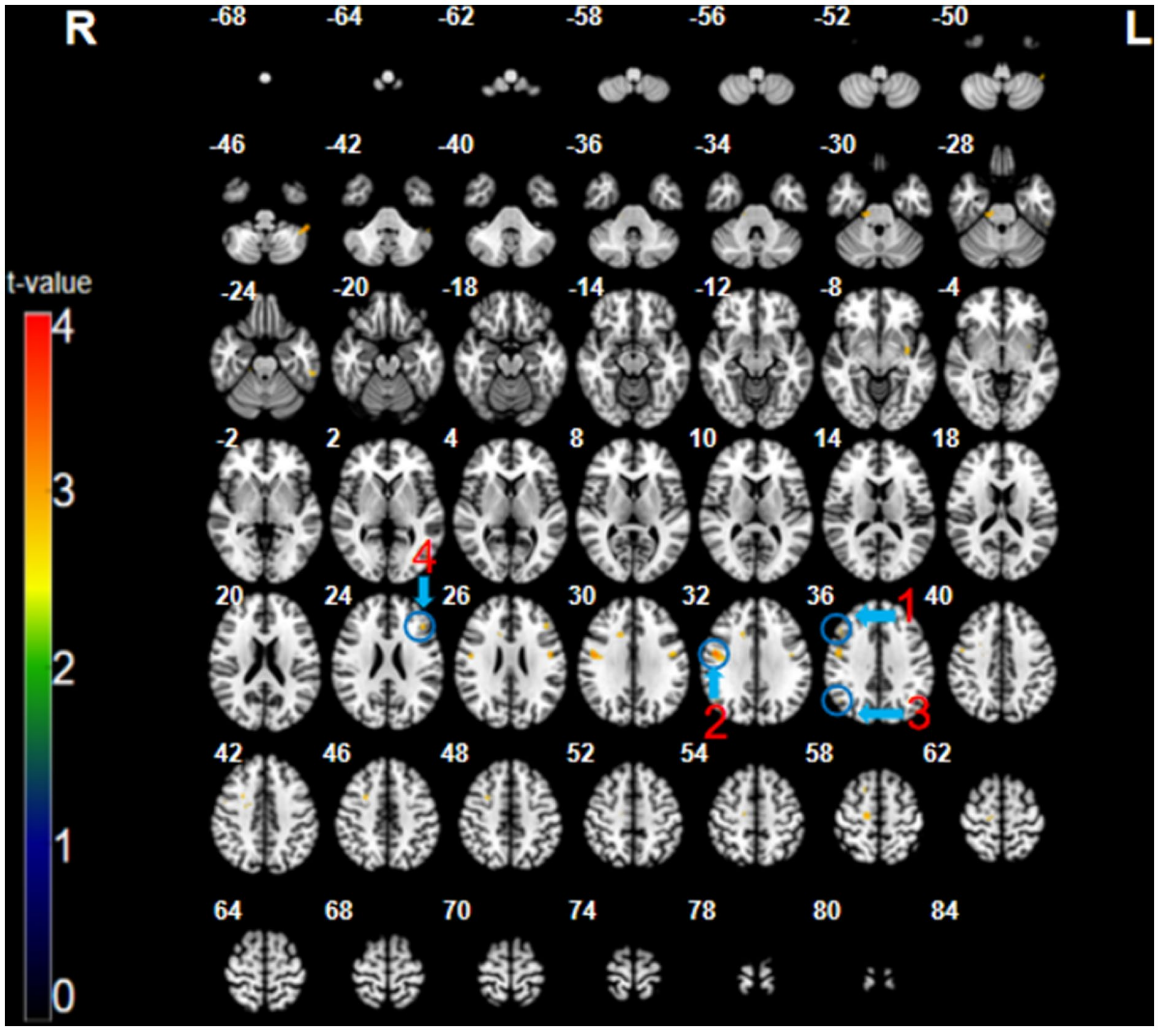
The CMT minus SMT contrast (uncorrected: *p* < 0.005, cluster 5). Regions circled in blue represent activated areas by CMT performance after subtraction of the SMT: ①dorsolateral prefrontal cortex (R); ② posterior parietal cortex (R); ③ premotor cortex (R); and ④ superior frontal cortex (L).

The participants exhibited significantly higher activation during the CMT relative to that during the SMT in the right dorsolateral prefrontal cortex (DLPFC; BA 9; *x* = 48, *y* = 26, *z* = 35, *t* = 3.16, *p* < 0.005, cluster size = 5 voxels), right posterior parietal cortex (PPC; BA 40; *x* = 48, *y* = −64, *z* = 35, *t* = 3.09, *p* < 0.005, cluster size = 6 voxels), right precentral gyrus (PMC; BA 6; *x* = 51, *y* = −4, *z* = 32, *t* = 3.73, *p* < 0.005, cluster size = 48 voxels), and the left superior frontal cortex (SFC; BA 46; *x* = −45, *y* = 29, *z* = 23, *t* = 3.26, *p* < 0.005, cluster size = 8 voxels) (Figure 2; Table 1).

**Table 1:** Regional cerebral activation in the contrast SMT < CMT and SMT < CT (uncorrected: *p* < 0.005, cluster 5).

Region		Cluster size	Coordinates (mm)			Brodmann area	Peak T
			*x*	*y*	*z*		
**SMT < CMT contrast**							
Dorsolateral prefrontal cortex	R	5	48	26	35	BA 9	3.16
Posterior parietal cortex	R	6	48	−64	35	BA 40	3.09
Precentral gyrus	R	48	51	−4	32	BA 6	3.73
Superior frontal cortex	L	8	−45	29	23	BA 46	3.26
**SMT < CT contrast**							
Caudate	L	7	−9	5	8		3.14
Medial prefrontal cortex	R	12	6	56	20	BA 9, 10	2.91
Primary somatosensory cortex	R	9	54	−19	53	BA 1, 3	3.75

### 3.2. CT minus SMT contrast

**Figure 3:**
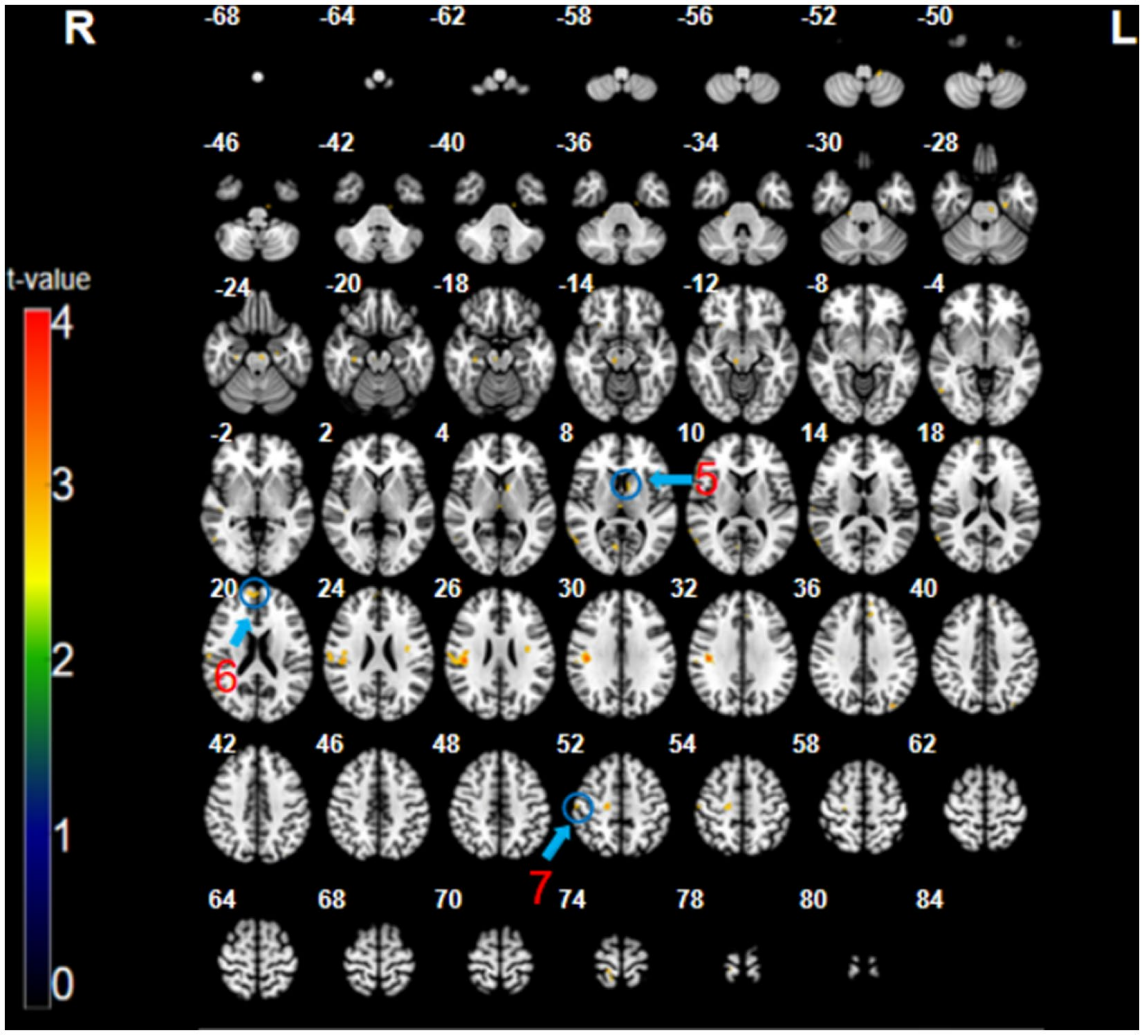
The CT minus SMT contrast (uncorrected: *p* < 0.005, cluster 5). Regions circled in blue represent activated areas by CT performance after subtraction of the SMT: ⑤ caudate (L); ⑥ medial prefrontal cortex (R); and ⑦ primary somatosensory cortex (R).

The analysis of the CT minus SMT revealed higher activation in the left caudate (*x* = 7, *y* = −9, *z* = 8, *t* = 3.14, *p* < 0.005, cluster size = 7 voxels), right medial prefrontal cortex (mPFC; BA 9, 10; *x* = 12, *y* = 56, *z* = 20, *t* = 2.91, *p* < 0.005, cluster size = 12 voxels), and the right postcentral gyrus (primary somatosensory cortex; BA 1, 3; *x* = 54, *y* = −19, *z* = 53, *t* = 3.75, *p* < 0.005, cluster size = 9 voxels) (Figure 3; Table 1). These brain regions were relatively more engaged by the CT than the SMT.

### 3.3. CT minus CMT contrast

The participants did not exhibit any significant differences in cerebral activation during CT performance after subtraction of CMT data (uncorrected: *p* < 0.005, cluster 5).

## 4. Discussion

This study aimed to determine the neurophysiological substrates underlying cognitive and motor skills by investigating the differences in brain activation. To this end, we observed the relative activation patterns in various brain regions for three types of tasks (SMT, CMT, and CT).

The CMT minus SMT contrast demonstrated activations in DLPFC, PPC, precentral gyrus, and SFC. The DLPFC is responsible for working memory (e.g., visuospatial information), motor planning, cognitive flexibility, and decision making (Petrides, 2000; Seo et al., 2007; Kaplan et al., 2016). Furthermore, the PPC is closely associated with visuospatial attention, decision making, and planned movement (Petersen and Posner, 2012; Yang et al., 2022). Specifically, the precentral gyrus, where the PMC is located, is mainly responsible for motor control, voluntary movement, motor preparation, motor sensation, and spatial sensation (Chen et al., 1995; Saito et al., 2019), whereas the SFC contributes to higher cognitive functions, particularly working memory and action selection (Courtney et al., 1998; Rushworth et al., 2004; Boisgueheneuc et al., 2006).

Accordingly, higher cerebral activation was observed during the CMT than that during the SMT, which was associated with increased visuospatial attention, working memory, and motor planning. The participants probably found the CMT more difficult than the SMT as a higher number of stones (25–35 for CMT vs. 4 for SMT) were placed on the grid. More optional spots to place the stone necessitates a greater cognitive effort to simultaneously evaluate and compare various options (i.e., visuospatial attention and working memory) to eliminate the wrong options and select the right spot to make *five in a row* (i.e., decision making) and to plan and execute the response (i.e., movement planning and execution). Furthermore, such cognitive processes might have been more strenuous considering the time limit (12 s) for performing the task, which might have impacted the functional activation of the recruited regions. Consistent with our findings, previous studies have also reported activation of the DLPFC during complex Gomoku tasks (Chen et al., 2003) and of the PPC during the use of selective or spatiotemporal attention (Behrmann et al., 2004). Yee et al. (2010) observed that the SFC was activated during tasks that involved the working memory for shape identification.

The activation of the PMC observed during the CMT in this study suggests that the participants made decisions on the spot using motor imagery to intentionally imagine their hands moving although no physical stones were used in the experiment. To mentally place a stone on the Gomoku grid during the CMT, the participants needed to use motor imagery to precisely control the direction of movement across a grid packed with black and white stones and ensure that the stone was placed in the intended coordinates. This process might have generated a motor sensation, thus activating the PMC. Buccino et al. (2001) considered the PMC to be associated with finger movements, and Ochiai et al. (2005) identified that it played a crucial role in controlling hand movements; together, these studies support our findings.

Interestingly, although the motor responses (mentally performed) required for the CMT and SMT were identical (i.e., placing a black stone), there was a higher activation of the brain regions responsible for motor planning and execution in the CMT. This finding suggests that the cognitive difficulty of the task not only affects stimulus processing but also neuronal activity related to motor responses.

The CT minus SMT contrast showed activation in the left caudate nucleus, right mPFC, and right S1. The caudate nucleus plays a key role in learning and performance. It is associated with learning, memory, feedback, reward, motivation, and emotion and helps process visuospatial information and control movement (Monchi et al., 2006; Grahn et al., 2008). The activation of the caudate nucleus during the CT but not during the SMT may indicate the recruitment of visuospatial working memory. Previous fMRI studies have reported caudate nucleus activation during route navigation tasks requiring visuospatial processing (Bohbot et al., 2007; Etchamendy and Bohbot, 2007). During a virtual maze task, individuals who navigated using a response strategy that repeatedly followed a specific route to find the way and avoid wrong turns demonstrated higher caudate activity (Bohbot et al., 2007). The participants performing the CT in our study might have used a similar response strategy by applying visuospatial working memory to identify the best spot to place the Gomoku stone on the grid, thus contributing to caudate activation.

In addition, the caudate activation during the CT compared with the SMT might be associated with the feedback system. This activation could be explained because during the CT of the Gomoku game, the participants tried to identify the best positions and received feedback on the advantages and disadvantages of the available positions from their internal evaluation. In fact, multiple studies have reported that the caudate responds to feedback during learning and may be a critical moderator of feedback influence (Tricomi et al., 2006; Bick et al., 2019).

The major roles of the mPFC in decision making include conflict monitoring (Botvinick et al., 2004), prediction (Alexander and Brown, 2011), error detection (Holroyd et al., 2002), and risk assessment (Bechara and Damasio, 2005). Gomoku is a strategic game that requires a high level of cognitive engagement; the players must think hard before placing their stones to reach the best decision and, ideally, win the game. Li et al. (2019) reported mPFC activation during decision tasks, which supports our interpretation. Another explanation for mPFC activation during the CT may be related to error detection. Participants facing the Gomoku board had to identify the correct location for the next move by evaluating several possibilities. During a trial-and-error task performance, the mPFC gets activated while detecting and rectifying the errors (Zarr and Brown, 2016). Therefore, the greater mPFC activation during the CT compared with that during the SMT in this study may reflect more complex decision making and error detection processing.

The S1 has been linked primarily to the processing of sensory information from the body as well as motor planning and production (Kropf et al., 2018). A recent study highlighted the role of the S1 in encoding imagined movement in the absence of sensory information (Jafari et al., 2020). Therefore, the S1 activation observed during the CT in this study affirms its role in cognitive imagery and engagement in the absence of sensation or expected sensation during motor production.

One explanation for the minimized activation during the SMT is that the participants had a minimum cognitive load as four stones were already placed on the board, thus enabling quick and easy responses. Witt et al. (2008) found reduced activation of the M1, SMA, and cerebellum during a simple tapping task compared with a complex task, which reinforces this explanation. Similarly, Nachev et al. (2008) observed higher SMA activation with increasing task difficulty. Schlerf et al. (2010) found that cerebellar activation was positively correlated with task complexity. Furthermore, the “memory drum” theory may support our explanation that complex tasks require more information storage and brain processing (and hence more time) than simple tasks that do not require the information-processing step, thus allowing a quick response (Henry and Rogers, 1960; Klapp, 2010).

This study has several limitations. In contrast to our initial prediction, no differences were observed between the CMT and CT. This could be attributed to the similar patterns of Gomoku stones presented in the CMT and CT despite the differences in the number of stones. Therefore, the participants performing the two tasks might have used similar strategies when searching for the optimal spot and making the corresponding decisions. Furthermore, as this experiment was conducted in an fMRI chamber, the motor tasks could not be performed externally. Thus, during the SMT and CMT, the participants were instructed to imagine placing a stone in an appropriate location, and during the CT, they were instructed to only strategically explore which location was appropriate for placing a stone without movement imagery. As the actual motor action did not occur, the characteristics of the motor task might not have fully appeared in the fMRI results; conversely, motor imagery might have naturally occurred even during the CT. Furthermore, due to the limited availability of participants and resources, only 30 participants were initially targeted, and ultimately, the data from 25 participants were used for analysis. A small sample size can potentially impact the reliability and generalizability of the conclusions drawn from the study. Therefore, in future studies, this study should be replicated with more participants to ensure that the results are consistent. Additionally, we recruited participants with minimal experience in Gomoku and familiarized them with the task for the experiment. However, we did not assess the degree of mastery of the task among the participants. Therefore, in future studies, additional measures such as a pre-test could be included to assess participants’ baseline skill levels and ensure that all participants have a similar level of experience with the task. Lastly, since this study has an exploratory nature, we analyzed the results using uncorrected value of ps to ensure that potentially important effects are not overlooked and to better understand the direction and magnitude of differences. In future studies, we recommend investigating the effects with a more conservative criterion using a corrected value of *p*.

In conclusion, we investigated the differences in brain activity during cognitive and motor tasks using fMRI. In the CMT minus SMT contrast, we found significant activation in the DLPFC, PPC, precentral gyrus, and SFC, which reflected increased visuospatial attention, working memory, and motor planning. In the CT minus SMT contrast, we observed significant activation in the caudate nucleus, mPFC, and S1, which are regions responsible for visuospatial working memory, error detection, and cognitive imagery, respectively. The present findings suggest that caution should be exercised in adopting traditional classification of cognitive and motor tasks, which simply focuses on the extent of decision making and motor control involved in task performance.

## Data availability statement

The raw data supporting the conclusions of this article will be made available by the authors, without undue reservation.

## Ethics statement

The studies involving human participants were reviewed and approved by the Kyungpook National University’s Institutional Review Board. The patients/participants provided their written informed consent to participate in this study.

## Author contributions

YL collected the data and performed the statistical analysis. YL and JK interpreted the results and wrote the first draft of the manuscript. TK contributed to the manuscript editing and revision. All authors contributed to the article and approved the submitted version.

## Conflict of interest

The authors declare that the research was conducted in the absence of any commercial or financial relationships that could be construed as a potential conflict of interest.

## Publisher’s note

All claims expressed in this article are solely those of the authors and do not necessarily represent those of their affiliated organizations, or those of the publisher, the editors and the reviewers. Any product that may be evaluated in this article, or claim that may be made by its manufacturer, is not guaranteed or endorsed by the publisher.

## Footnotes

^1^ http://www.fil.ion.ucl.ac.uk/spm/
