# Supplementary material for: A neurophysiological approach to the distinction between motor and cognitive skills: a functional magnetic resonance imaging study
Source: Front Neurosci. 2023 May 19;17:1178800. doi: 10.3389/fnins.2023.1178800 (PMC10235625; doi:10.3389/fnins.2023.1178800)
Supplement: Supplementary file 1 [file Data_Sheet_1.docx]

Supplementary Material

A neurophysiological approach to the distinction between motor and cognitive skills: a functional magnetic resonance imaging study

Yunhang Lu, Jingu Kim, and Teri Kim*

*** Correspondence:** Corresponding Author: terikim@knu.ac.kr

# Supplementary Figures and Tables

## Supplementary Figures


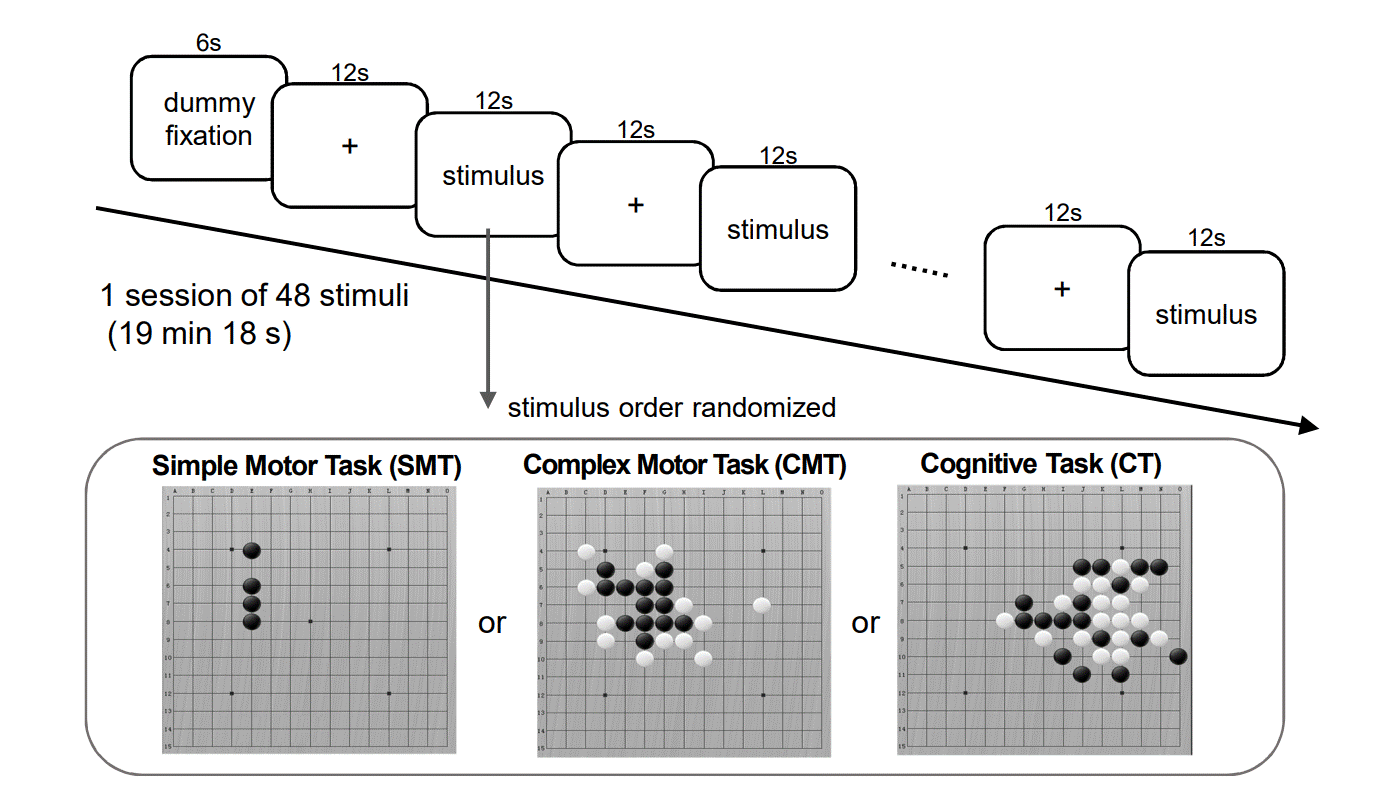


**Supplementary Figure 1.** The experimental paradigm for the study

## Supplementary Tables

Supplementary table 1. Regional cerebral activation during SMT, CMT, and CT tasks

| Task | Region |  | Cluster size  (voxels) | MNI Coordinates (mm) | | | Brodmann Area | Peak T |
| --- | --- | --- | --- | --- | --- | --- | --- | --- |
|  |  |  |  | x | y | z |  |  |
| SMT | Fusiform gyrus | L | 112 | −24 | −70 | −4 | BA19 | 5.91 |
|  |  | R | 163 | 24 | −70 | −13 | BA19 | 5.73 |
|  | Lingual gyrus | L | 288 | −15 | −76 | −7 | BA18 | 7.39 |
|  |  | R | 286 | 15 | −76 | −7 | BA18 | 7.49 |
|  | Middle occipital cortex | L | 309 | −30 | −91 | 20 | BA19 | 5.48 |
|  |  | R | 208 | 30 | −88 | 20 | BA19 | 5.78 |
|  | Superior parietal cortex | L | 200 | −21 | −67 | 50 | BA7 | 4.11 |
|  |  | R | 91 | 27 | −64 | 50 | BA7 | 3.66 |
|  | Precentral | L | 205 | −30 | −7 | 50 | BA6 | 4.16 |
|  |  | R | 21 | 27 | −4 | 50 | BA6 | 3.95 |
|  | Supplementary motor area | L | 56 | −9 | 2 | 68 | BA6 | 4.51 |
|  |  | R | 37 | 10 | −3 | 68 | BA6 | 3.93 |
| CMT | Fusiform gyrus | L | 270 | −24 | −76 | −10 | BA18 | 12.67 |
|  |  | R | 291 | 24 | −76 | −10 | BA18 | 9.67 |
|  | Lingual gyrus | L | 332 | −18 | −76 | −10 | BA18 | 10.01 |
|  |  | R | 339 | 9 | −73 | −7 | BA18 | 7.45 |
|  | Middle occipital cortex | L | 530 | −30 | −91 | −1 | BA18 | 7.51 |
|  |  | R | 200 | −33 | −88 | 2 | BA19 | 8.52 |
|  | Superior parietal cortex | L | 387 | −18 | −64 | 56 | BA7 | 6.28 |
|  |  | R | 336 | 21 | −64 | 56 | BA7 | 7.04 |
|  | Precentral | L | 421 | −52 | 4 | 44 | BA8 | 6.29 |
|  |  | R | 48 | 54 | 11 | 35 | BA9 | 4.75 |
|  | Supplementary motor area | L | 198 | −9 | 5 | 62 | BA6 | 4.57 |
|  |  | R | 141 | 12 | 5 | 62 | BA6 | 4.40 |
|  | Superior frontal cortex | L | 214 | −24 | −1 | 62 | BA6 | 9.51 |
|  |  | R | 232 | 24 | 2 | 59 | BA6 | 8.47 |
|  | Caudate | L | 38 | −9 | 8 | 11 |  | 3.67 |
|  |  | R | 46 | 9 | 8 | 11 |  | 2.83 |
|  | Thalamus | L | 9 | −9 | −19 | 11 |  | 4.25 |
|  |  | R | 9 | 12 | −19 | 11 |  | 5.50 |
|  | Insula | L | 84 | −30 | 23 | 5 |  | 6.54 |
|  |  | R | 52 | 33 | 20 | 5 |  | 3.70 |
| CT | Fusiform gyrus | L | 268 | −24 | −76 | −10 | BA18 | 12.88 |
|  |  | R | 289 | 24 | −76 | −10 | BA18 | 10.32 |
|  | Lingual gyrus | L | 332 | −21 | −73 | −10 | BA18 | 11.75 |
|  |  | R | 334 | 6 | −73 | −1 | BA18 | 8.17 |
|  | Middle occipital cortex | L | 563 | −30 | −91 | −1 | BA18 | 8.27 |
|  |  | R | 325 | −33 | −88 | 2 | BA19 | 9.02 |
|  | Superior parietal cortex | L | 390 | −18 | −64 | 56 | BA7 | 6.65 |
|  |  | R | 318 | 21 | −64 | 56 | BA7 | 7.69 |
|  | Precentral | L | 353 | −52 | 4 | 44 | BA8 | 4.83 |
|  |  | R | 78 | 54 | 11 | 35 | BA9 | 4.55 |
|  | Supplementary motor area | L | 116 | −9 | 5 | 62 | BA6 | 3.72 |
|  |  | R | 74 | 12 | 5 | 62 | BA6 | 3.39 |
|  | Superior frontal cortex | L | 192 | −24 | −1 | 62 | BA6 | 9.49 |
|  |  | R | 230 | 24 | 2 | 59 | BA6 | 8.04 |
|  | Caudate | L | 38 | −9 | 8 | 11 |  | 4.86 |
|  |  | R | 46 | 9 | 8 | 11 |  | 5.80 |
|  | Thalamus | L | 9 | −9 | −19 | 11 |  | 6.29 |
|  |  | R | 9 | 12 | −19 | 11 |  | 6.64 |
|  | Insula | L | 84 | −30 | 23 | 5 |  | 7.53 |
|  |  | R | 52 | 33 | 20 | 5 |  | 5.61 |

Note. R = Right; L = Left; SMT = simple motor task; CMT = complex motor task; CT = cognitive task
